# Carbon Nanocomposites in Aerospace Technology: A Way to Protect Low-Orbit Satellites

**DOI:** 10.3390/nano13111763

**Published:** 2023-05-30

**Authors:** Janith Weerasinghe, Karthika Prasad, Joice Mathew, Eduardo Trifoni, Oleg Baranov, Igor Levchenko, Kateryna Bazaka

**Affiliations:** 1 School of Engineering, College of Engineering, Computing and Cybernetics, The Australian National University, Canberra, ACT 2600, Australia or levchenko.igor@nie.edu.sg (I.L.); katia.bazaka@anu.edu.au (K.B.); 2 Advanced Instrumentation and Technology Centre, Research School of Astronomy & Astrophysics, ANU College of Science, The Australian National University, Canberra, ACT 2600, Australia; 3 Department of Theoretical Mechanics, Engineering and Robomechanical Systems, National Aerospace University, 61070 Kharkiv, Ukraine; 4 Department of Gaseous Electronics, Jozef Stefan Institute, 1000 Ljubljana, Slovenia; 5 Plasma Sources and Application Centre, NIE, Nanyang Technological University, Singapore 637616, Singapore

**Keywords:** carbon nanomaterials, LEO satellites, satellite corrosion, spacecraft, space environment, nanotechnology

## Abstract

Recent advancements in space technology and reduced launching cost led companies, defence and government organisations to turn their attention to low Earth orbit (LEO) and very low Earth orbit (VLEO) satellites, for they offer significant advantages over other types of spacecraft and present an attractive solution for observation, communication and other tasks. However, keeping satellites in LEO and VLEO presents a unique set of challenges, in addition to those typically associated with exposure to space environment such as damage from space debris, thermal fluctuations, radiation and thermal management in vacuum. The structural and functional elements of LEO and especially VLEO satellites are significantly affected by residual atmosphere and, in particular, atomic oxygen (*AO*). At VLEO, the remaining atmosphere is dense enough to create significant drag and quicky de-orbit satellites; thus, thrusters are needed to keep them on a stable orbit. Atomic oxygen-induced material erosion is another key challenge to overcome during the design phase of LEO and VLEO spacecraft. This review covered the corrosion interactions between the satellites and the low orbit environment, and how it can be minimised through the use of carbon-based nanomaterials and their composites. The review also discussed key mechanisms and challenges underpinning material design and fabrication, and it outlined the current research in this area.

## 1. Introduction 

**Figure 1:**
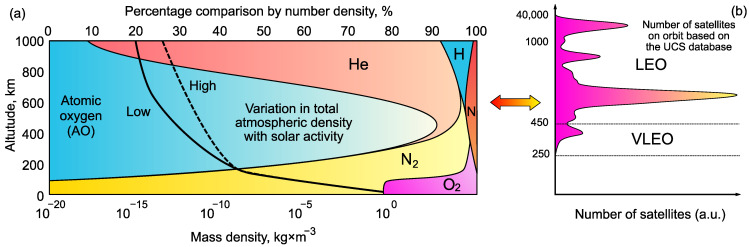
(**a**) Atmospheric density and composition at different altitudes. The highly reactive atomic oxygen (*AO*) is the predominant chemical species in VLEO. Data from N.H. Crisp et al., 2022 [2]. (**b**) Number of satellites on orbit based on the database of UCS. Data from J. Wu et al., 2022 [11] © 2022 Elsevier.

Earth orbits are mainly classified into very low earth orbits (VLEO), low earth orbits (LEO), medium earth orbits (MEO) and high orbits. VLEO orbits are the closest (starting from approximately 100 km) to the Earth, while LEO are currently the most populated orbits located at an altitude range spanning 200 to about 2000 km [1,2] (Figure 1). The LEO satellites have high velocity (>25,000 km/h) because of this low altitude, and it takes them less than two hours to complete a single orbit around the Earth [3].

LEO can be used for communication by networking multiple satellites together [4,5]. These networks are also known as LEO constellations. Iridium, Globalstar and Starlink are examples of such satellite networks [6]. Due to the shorter distance between the ground stations and LEO, the signal latency is comparatively low (5–10 ms) [7]. Transmission power of LEO satellites can also be kept low due to this short distance. Another advantage of LEO is lower launching costs due to a significantly lower impulse needed to launch satellites to lower orbits. Other uses for LEO include the global positioning system (GPS), space environment studies, space stations and technology demonstrations. The growing commercial space economy is particularly heavily concentrated on LEO, with the proposed expansion of Starlink constellation aiming to deliver up to 42,000 communication satellites for internet-based services [8].

The use of VLEO satellites offers even greater advantages as compared with LEO spacecraft and, for this reason, there is currently an increasing tendency for satellite constellations to occupy lower orbits. To compare, the Iridium telecom constellation (maiden launch in 1997) utilizes the orbits of near 870 km, while SpaceX plans to lower the orbit of newly launched satellites to 340 km. EOI Space plans to occupy an even lower shell, designing its “Stingrays” satellites for ultra-high-resolution imagery, and making them capable of operating in a relatively dense atmosphere [9]. In the European Union, a “Horizon 2020” program for “radical redesigning” of satellites is underway, with the aim of targeting lower orbits [10]. Indeed, very low orbits offer specific advantages including (i) smaller, cheaper optical systems for ultra-high-resolution imagery; (ii) enhanced resolution for the available optical systems; (iii) lower requirements for power for data transmission, (iv) higher signal-to-noise ratio; (v) the possibility of using aerodynamic surfaces for manoeuvring [2]; and (vi) of specific importance, a significantly lower latency in data transfer between satellites and the Earth. Low signal delay enables many important features that can not be readily realized when using high-delay networking. 

However, nothing comes for free: low orbits are located in the near-Earth layer where the residual atmosphere is much denser compared to higher orbits (Figure 1a). This presents two key problems for the use of low-orbit satellites—the velocity loss due to atmospheric drag and the erosion of materials under action of residual atmosphere. Nevertheless, LEO remains the most occupied orbital range for satellites (Figure 1b allows to directly compare the distribution of satellites by altitudes and the density of residual atmosphere).

**Figure 2:**
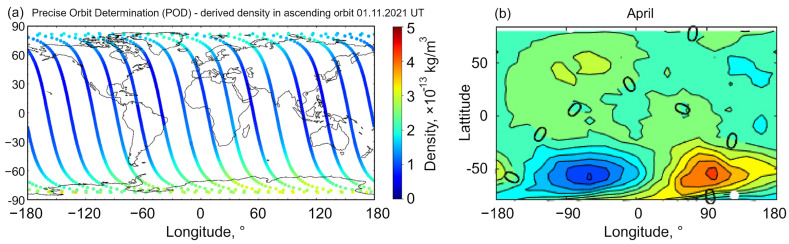
Atmospheric density around the globe is not a constant value. (**a**) Atmospheric density measured by the spherical Qiu Qiu (QQ) satellite. The density changes by a factor of 3–4 during, thus potentially representing a significant disturbance for satellites occupying these orbits. Reprinted from Y. Sun et al., 2022 [14] under terms and conditions of the CC BY license. (**b**) Along with the global un-uniformity of the atmospheric density, it also changes with the seasons due to differences in solar radiation. Reprinted from G. Chen et al., 2023 [13] under terms and conditions of the CC BY license.

Apart from other low orbits, there are two more important types of orbits that need to be traced through the high-density atmosphere: the polar and near-polar orbits, and the so-called super low perigee orbit, SuLPO. The polar and near-polar orbits are of great importance, as they allow for Sun-synchronous orbiting, when the satellite passes any specific point at the Earth surface at the same local time. This is imperative for many applications but requires very low orbiting for the precise synchronization. However, along with the higher residual gas density, low polar orbits face one more challenge, namely a significant change in the atmospheric density at the North and South poles, due to the Earth’s magnetic field. Moreover, the change in the atmospheric density due to the difference in diurnal and nocturnal solar radiation with longitude is also significant [12]. Figure 2a illustrates variations in the atmospheric density measured by the satellite, and Figure 2b shows the density map for the specific month. Significantly different maps were obtained for other seasons [13]. The SuLPOs are elliptical orbits with high apogee and low perigee where satellites dip very close to the Earth’s surface, e.g., for ultra-high-resolution imaging, and they then leave low altitudes for the remainder of the orbital path. Satellites utilising SuLPO will also be subject to the effect of atmosphere.

✓ Thus, three factors affect the satellite-atmosphere interaction: orbit inclination, solar activity, and flight altitude. This interaction evokes two problems: velocity loss and material erosion. Under certain circumstances, these two problems become tightly interrelated, as in the case of VLEO satellites that use residual atmosphere as propellant for orbit-rising thrusters.

This interesting interrelation comes from the need to use thrusters to compensate for the velocity loss via atmospheric drag. Here, atmospheric gas, if properly collected and ionised, could be used, in principle, as propellant for electric propulsion thrusters powered by solar panels. As propellant carried on board of satellites contributes most to the total mass of a typical thrust systems and is a finite resources that greatly limits the satellite operational lifetime, the use of atmospheric-breathing thrusters for LEO and especially VLEO offers a great advantage, allowing for propulsion without propellant stored on board—and hence, the life cycle of such a solar-powered satellite could be very long.

**Figure 3:**
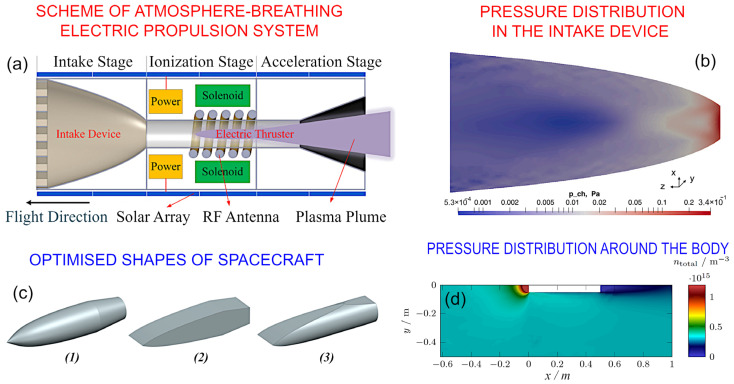
Residual atmosphere at VLEO inside and outside of a satellite. (**a**). Intake device collects the rarefied gas and supplies it to a thruster. The system can also include a thermalization chamber between the intake device and the ionization chamber of the thruster (not shown in this scheme). Reprinted with permission from P. Zheng et al., 2022, [23]. (**b**) Pressure distribution in the intake device at an altitude of 150 km. A case when the focal point of parabola is located inside the discharge channel of the thruster. The pressure rises significantly in some areas around the satellite part, and oxygen could cause significant damage, in particular in hot areas of the intake devices and thrusters. Reprinted with permission from F. Romano et al., 2021, [24]. External geometry of VLEO satellites also needs to be optimised to decrease drag. At the VLEO altitude range, the gas is rarefied and the free molecular flow should be considered, when the mean free pass of particles exceeds the size of a satellite. (**c**) Illustrates the three types of optimised shapes (marked ‘1’, ‘2’, and ‘3’) of spacecraft, and (**d**) shows the simulated pressure distribution around the body for the typical VLEO conditions. A significant increase in the particle densities over some areas could cause enhanced material damage. Reprinted with permission from F. Hild et al., 2022, [25]. © 2022 IAA. Published by Elsevier.

Figure 3a illustrates a typical system with intake devices, supply lines, an ionization chamber and a plasma acceleration system. It should be noted here that gas density at VLEO is significantly higher than at LEO; yet, it is still a free molecular flow, and so, it requires specific approaches for designing the intake devices. Figure 3b shows the calculated distribution of pressure in the intake device, and the pressure rise covers three orders of magnitude.

✓ The effect of atomic oxygen (*AO*) on the material in the intake devices and thruster could be a detrimental and life-limiting factor.

An increase in pressure around the satellite is one more factor of concern, and the satellite shape needs to be optimised to reduce drag—in a similar way to the shape of an aeroplane body. Figure 3c,d show the optimised shapes for VLEO satellites and the pressure distribution around the satellite body, evidencing a significant pressure rise and hence, a significant rise in *AO* fluence to the same parts of the spacecraft.

✓ VLEO and LEO satellites offer many significant advantages yet at the same time face several significant challenges; with the present-day tendency to refocus many of the key missions to low orbits, among other problems of operating at these orbits, issues pertaining to material degradation and stability under these conditions must be properly addressed so as not to hinder future development of low orbit satellite technology.

Research and development of space grade materials have a long history dating back to the beginning of space age. Presently available space materials were optimised to withstand the harsh conditions of the space environment, such as the effects of ultra-high vacuum, ionizing radiation, charge accumulation, UV radiation, thermal cycling and many other factors [15,16]. General application requirements also need to be fulfilled, such as weight reduction, mechanical stability, chemical reactivity, and cost reduction. Now, when the LEO and VLEO satellites tend to occupy much lower orbits, special attention should be paid to the specific material–*AO* interaction, and novel materials capable of operating for years in aggressive *AO*- enriched conditions at low orbits need to be designed.

Nanomaterials have at least one dimension within the range of 100 nm or less, and they often demonstrate outstanding mechanical, electrical, thermal and optical properties. The replacement of conventional space materials with those based on nanomaterials and nanocomposites can significantly improve many important characteristics of the related structural and functional spacecraft components [17].

Graphene, fullerene, carbon nanotubes (CNT), nanofibers, nanoclays and others are examples of carbon-based nanomaterials used in space applications [18]. The advanced properties of carbon nanomaterials are due to the properties of carbon itself, as well as the dimensional effects resulting from the nanoscaled sizes of elements and structures present within these nanomaterials. The general properties of carbon in the context of structural materials are quite diverse due to many possible allotrope forms, and different allotropies demonstrate very different chemical and mechanical properties. From graphite (consisting of layers of graphene) which is soft enough to be used in pencils to diamond which is among the hardest materials in the Universe—this impressively wide range of properties is directly attributed to the existence of multiple carbon allotrope forms. Electric conductivity of carbon allotropes also varies across a very wide range, from high conductivity of graphite which is used for electrodes to low conductance of diamond. On the other hand, thermal conductivity of diamond and graphene-based allotropes exceeds that of any other known material. In terms of mechanical strength, graphene is considered as the strongest material in the Universe, with the tensile strength reaching 130 GPa [19,20].

Not surprisingly, this unique family of materials attracted the attention of many researchers targeting various engineering problems, including protection from aggressive environments ranging from, e.g., marine fouling [21] to space propulsion thrusters and spacecraft [18,22]. The purpose of the present work is to briefly discuss the key mechanisms responsible for material degradation under the conditions of low orbit environment. In-flight and simulated experiments using carbon-based nanomaterials and their key findings, research trends and key challenges are also described.

## 2. Corrosion Factors and Mechanisms in Low Orbit Environment

The low orbit space is a dynamic environment that varies with different factors such as altitude, orbital position and solar activity. The effects of low orbit environment on spacecraft materials are highly important to determine their functionality and durability. These effects were studied in various in-flight and simulated space environments. Such experiments and their key findings are briefly described in this section.

### 2.1. LEO Environmental Challenges and Material Requirements

**Figure 4:**
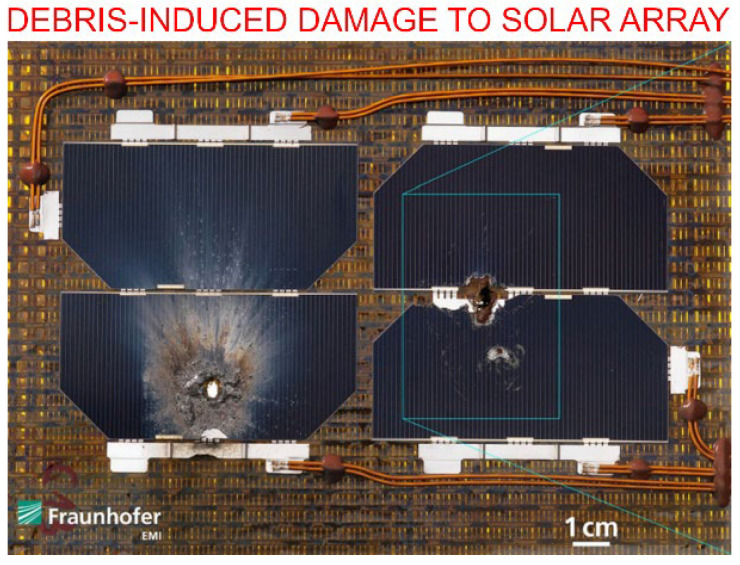
The impact of the collision with 1.59 mm aluminium spheres on the integrity of solar arrays. The experiment used sphere velocities typically detected in the collisions between space debris and satellites. Reprinted with permission from H. Krag et al., 2017 [32]. © 2017 Elsevier.

The harsh conditions of the low orbit environment typically lead to premature mechanical, thermal and chemical degradation of the spacecraft materials. The effects from space radiation (cosmic rays, solar wind, UV), thermal cycles, vacuum environment and debris need to be considered [26,27]. Space debris materials and natural micrometeoroids also represent a serious hazard to the external surfaces of spacecrafts, since the relative velocity of these materials can reach tens of kilometres per seconds. Collisions with these space debris could be responsible for complete failure or malfunction of the entire satellite or its subsystems [28]. An example of such damage is shown in Figure 4.

The UV radiation at low orbit contains two components, namely the vacuum UV at the wavelength of 100–200 nm and the near field UV in the 200–400 nm range. About 8% of the total solar radiation is due to the UV radiation and it causes measurable chemical and mechanical changes in spacecraft materials [29]. UV radiation effects include bond breaking in functional groups such as C–C, C–O, etc., modification of polymers, mass loss of materials by creation of volatile fragments and others. Radiation levels at LEO are around 300 µGy and it could go up due to solar events [30]. These effects lead to material degradation and loss of their initial thermal, electrical and mechanical properties [31].

Thermal cycling is one more problem intrinsic to orbital environment; yet, for low orbits, the thermal cycle takes place within a few hours. Thermal fatigue due to this repeated rapid thermal stress ultimately degrades the mechanical properties of spacecraft materials. Therefore, thermal cycling is an important factor to be consider on material selection for LEO environment.

Ionizing radiation of the LEO environment is mainly due to galactic cosmic rays, solar activity and radiation belts. This includes alpha particles, high energy beta particles and gamma radiation. Incident ionizing radiation may affect material properties and often results in degradation effects via ionization, atomic displacement, bond breaking, etc. The intensity of these adverse effects depends on the type of radiation, energy and dose.

*AO* (atomic oxygen) is the most abundant gaseous species in LEO which is formed due to photo-dissociation of molecular oxygen at low earth altitudes (Figure 1a). The recombination of dissociated oxygen atoms is unlikely due to the high energy environment and longer mean free paths [33]. Other species include high energy protons (H nuclei), nitrogen, oxygen molecules and argon. At the altitude of 300 km, 80% of gaseous species are *AO* and 20% is composed of nitrogen molecules, with the *AO* density varying from about 10^8^ to (2–4) × 10^9^ atoms × cm^−3^, depending on the level of solar activity [34]. The exact flux value is determined by various factors such as altitude, orbital inclination, solar activity and time of day. Despite the low number of species present, high velocities of spacecraft provide a translational energy of around 5 eV per *AO* atom, which is sufficient to cause oxidative decomposition and bond breaking in polymers [35,36]. Interactions with *AO* cause surface erosion, chemical changes, morphological changes, the formation of particulates, material contamination and space vehicle glow which interfere with the function of optical sensors [31].

Charge prevention or minimization is another aspect which needs to be considered when undertaking the design of spacecraft materials. Since polar regions are characterized by low-density high energy plasmas and other regions can contain low energy plasmas, spacecraft can accumulate a static electric charge that could interfere with the function of spacecraft electronic systems. To prevent this, surface properties of the material such as electrical resistivity, secondary electron emissivity and photoelectron emissivity should be properly selected [33].

Thus, superior tensile strength, higher thermal stability and outstanding chemical resistance are among the key factors that need to be considered during the selection of materials for low orbit satellites. Some other properties, e.g., electron emissivity, could also be important.

### 2.2. Simulated Corrosion Experiments

In-flight experiments on space materials are very expensive and require a long time for preparation and analysis. In this light, simulations and theoretical investigation are very important for designing new materials for space applications. Many researchers studied *AO*-induced degradation of various materials in simulated space environments. Chen et al. conducted decay tests on Poly(p-phenylene benzobisoxazole) (PBO) fibres which are used in tether cables and composite reinforcements. Morphological studies indicated severe roughening and erosion of the fibres and mechanical studies revealed a significant decrease in tensile strength retention ratios [37]. Liu et al. reported the bending strength loss and surface resistivity changes on zirconium carbide coated carbon composites [35]. Cyanate-based shape memory polymers were shown to undergo an accelerated aging, by peeling off the material’s outer layer after reacting with *AO*. However, shape memory behaviour, thermal stability and mechanical properties were not significantly affected after the experiment. Interestingly, the reactions with *AO* resulted in the formation of new carbonyl and hydroxyl bonds within the polymer, with new peaks detected in the FTIR spectra [36]. A similar study was conducted by Wang et al. by introducing carbon fibre into the cyanate ester for extra durability [38]. Gotlib-Vainstein et al. studied the stability of TiO_2_- and SnO_2_-based coating materials against *AO* erosion [39]. Polytetrafluoroethylene (PTFE), another polymeric material often used in spacecraft, showed mass loss, changes in chemical properties and optical properties due to *AO* reactions. However, thermal properties were unaffected according to the TGA studies.

**Figure 5:**
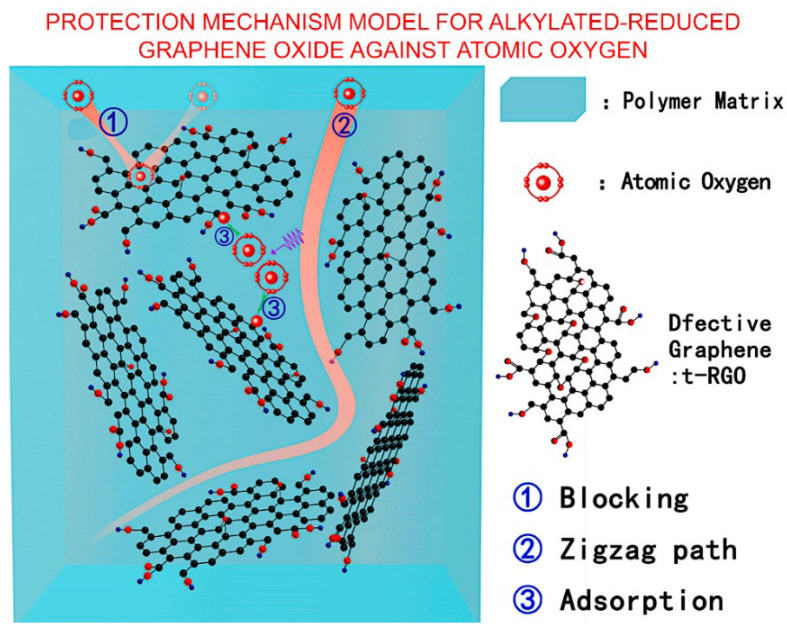
Proposed mechanism by which alkylated-reduced graphene oxide additive affords protection against *AO* damage to a polymer matrix. The uniform dispersion of ultra-high modulus RGO in the polymer matrix can effectively mitigate the impact of high-energy *AO*, which effectively protects the polymer matrix from direct impact (route 1). Reprinted with permission from Xie et al., 2015 [40] © 2015 Elsevier.

An example of the efficient application of simulation-based research is illustrated in Figure 5. Xie et al. conducted experimental studies and DFT simulations on reduced graphene oxide (RGO)-enhanced polypropylene composites. Theoretical studies indicated that defective graphene shows lower binding energy with *AO* than pure graphene. It was experimentally investigated and 0.5 wt% of RGO loading found ideal for enhanced mechanical properties [40].

✓ Simulation and theoretical studies are very important in the design of space materials, and novel approaches including those based on artificial intelligence (AI) and machine learning (ML) need to be implemented to support fast progress in this field.

### 2.3. In-Flight Corrosion Experiments

More than 1000 materials were tested in flight by several space missions. Space shuttle missions (STS-5, STS-8, STS-17, STS-32, STS-41, STS-46) and satellites (LDEF, Solar Max, COMES experiment) were used to select suitable materials for future space missions [31].

The Materials International Space Station Experiment (MISSE) project is an extensive in situ long-term study conducted by NASA to study the effect of LEO environment on various materials. The samples were mounted externally on the international space station and were subjected to the effects of LEO environment [29]. Suitcase-like trays known as passive experiment containers were exposed to the space environment on both ram and wake directions of the International Space Station. It was found that the *AO* erosion was two orders of magnitude grater in the ram direction than that in the wake direction due to the high impact energies in the former [41]. Some of the key results of the MISSE program are summarized in Table 1.

**Figure 6:**
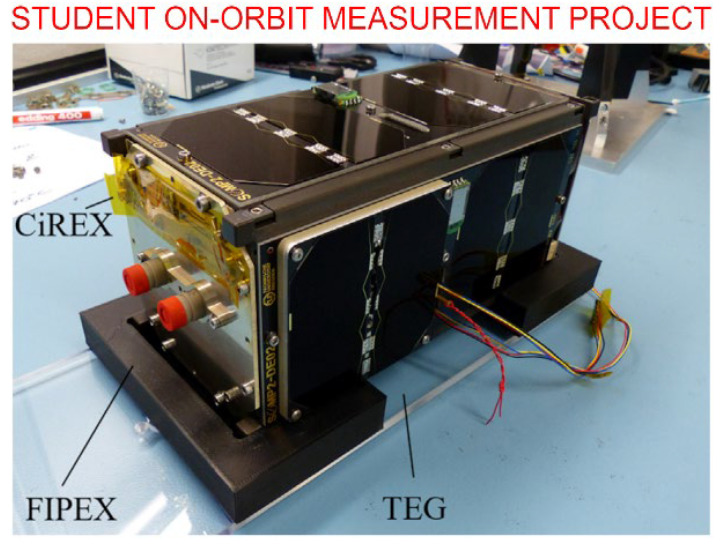
In-orbit material experiment project. Based on a 2U (double-unit) CubeSat, this system incorporates several material experiments, namely CiREX (Carbon Nanotubes—Resistance Experiment), Flux-(Phi)-Probe-Experiment (FIPEX) and Thermoelectric-Generator-Experiment (TEG). Reprinted with permission from Abbe et al., 2019 [43]. © 2018 COSPAR. Published by Elsevier.

An example of the recent experiments is shown in Figure 6. Here, a 2U Cubesat platform was used to prepare flight experiments to investigate the effect of exposure to space environment on different materials.

### 2.4. Lab-Based AO Corrosion Experiments and Facilities

It should be noted that, to some extent, both simulated and in-flight corrosion experiments can be either not very reliable, or very expensive ways to optimize the corrosion behavior and decrease the impact of corrosion on space assets. In contrast, when properly designed, relevant experiments in conventional laboratories dealing with material science would be of great help when it comes to collecting large volumes of potentially valuable experimental data on space-related corrosion, while providing a more rapid path to optimization and innovation. Indeed, LEO degradation and erosion are simulated in ground laboratories using a variety of experimental facilities, each with their own limitations. These include exposure to RF plasma, low-energy ion neutralization, electron-stimulated desorption, photodissociation, supersonic and laser detonation sources [44].

Currently, extensive efforts are applied to develop more sophisticated, well-equipped ground-based test facilities for comprehensive testing of space-intended materials under the conditions specific to LEO and VLEO orbits. The LEO Atomic Oxygen Interaction Facility at the Australian National University, National Space Test Facility (NSTF) in Canberra, Australia, is an example of such efforts. Recently reported results of the atomic oxygen interaction experiments [45] were related to the assessment of the *AO* effect on silicone, with the nanoindentation technology used to characterize the mechanical properties of the material before and after *AO* exposure. Further development of this facility is planned to test various materials under space conditions, with the carbonous materials, e.g., such as carbon fibres, being in focus. Using this and similar set-ups, it will be possible to effectively and rapidly observe the potential change in materials characteristics under the effect of *AO* exposure without highly expensive space flight tests, greatly enhancing our ability to explore other more innovative materials and material architectures at a relatively low cost.

**Figure 7:**
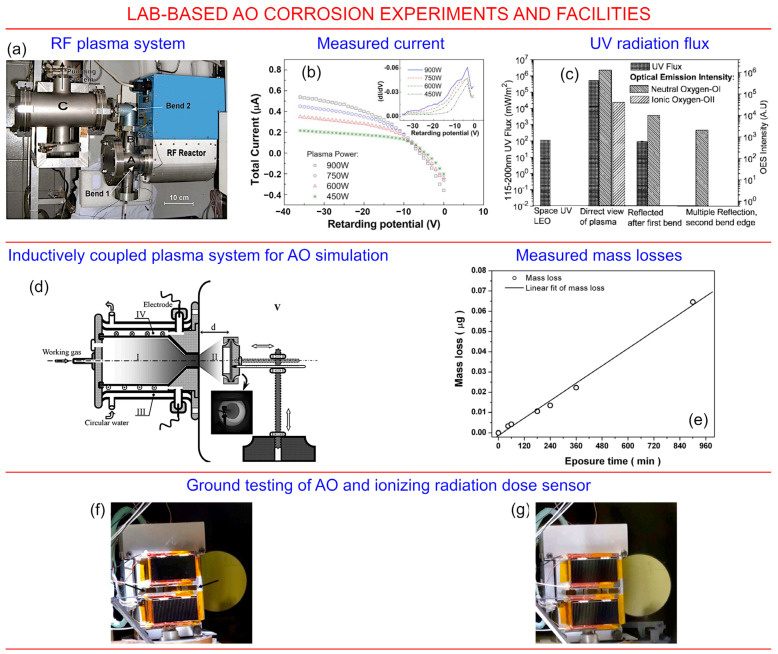
Lab-based *AO* corrosion experiments and facilities. (**a**) Radio-frequency system. (**b**) The measured current is dependent on the negative bias (inset illustrates the distribution of electron energy). (**c**) The ultra-violet flux and optical emission spectroscopy (OES intensity). Reproduced with permission from Shpilman et al. [49]. © AIP. (**d**,**e**) Scheme of the experiment and the measured mass loss due to *AO* exposure. The designed system ensures regulation of the *AO* flux, thus enabling modelling the *AO* impact at various altitudes. Reproduced with permission from Huang et al. [50]. Copyright AIP. (**f**,**g**) The ground test of a space materials degradation detector before and after exposure to an *AO* flux. Reproduced with permission from Verker et al., 2020 [51]. © Elsevier.

Radio-frequency (RF) plasma technology is among the most promising techniques for designing the on-ground test facilities for *AO*-material interaction studies. Figure 7a–c illustrate an approach to build a thermal atomic oxygen source using a radio-frequency plasma system. The authors interpreted the decrease in ion current and ultra-violet flux as the electron flux from the metal walls of the test chamber, along with the absorption of the ultra-violet flux. Apparently, these factors would be absent under the real space conditions; thus, the process of building specific-conditions test facilities that would adequately reflect the complexity of different space environments represents a rather significant engineering challenge.

Another approach to ground testing of space-related materials is based on the application of inductively coupled plasma systems, which are quite common in materials research. Figure 7d,e shows a scheme of an experiment and the measured mass loss due to the *AO* exposure. In this system, the authors reported a stable operation of this novel system, with the capability to support long-term tests with a highly regulated flux of atomic oxygen.

Figure 7f,g illustrates the ground tests of an *AO* flux and ionizing radiation dose sensor. This is an example of the recent level of the ground tests, the aim of which was to predict the degradation of materials by the action of *AO* in space environment. In this approach, the *AO* flux was measured by detecting a change in the behaviour of thin layers of organic materials. These and other efforts focused on the design of novel, sophisticated material degradation test systems are likely to continue to gather momentum [46,47,48].

### 2.5. Corrosion Mechanisms 

Among the different types of erosion, the *AO*-induced corrosion is particularly detrimental to satellites, since it may affect the key elements supporting the function and longevity of satellites, such as antennas and the energy system. Not surprisingly, significant efforts were made to develop effective protection for these critical elements.

Morphological studies on Poly(p-phenylene benzobisoxazole) (PBO) fibres showed severe erosion upon the *AO* exposure. The deterioration of crystal structure was observed during the XRD studies and XPS revealed a decrease in the peak corresponding to C–C bond, attributed to the oxidation of material by *AO* [37]. Additionally, a slight decrease in N components and a significant increase in O components were reported in polyamide-imide (PAI)/Polytetrafluoroethylene (PTFE) composite coatings [52]. The erosion reaction mechanism of *AO* with carbon can be given by Equation (1) [35]. The erosion yield of a material is dependent on the chemical nature of the material such as the carbon content, degree of aromaticity, etc. [53].

(1) $$C_{\left(s\right)}+{3AO}_{\left(p\right)}\to {CO}_{2(g)}+{CO}_{\left(g\right)}.$$

More than 90% of the product flux can be attributed to the formation of *CO* and *CO*_2_. Additionally, the formation of water and OH was observed via mass spectrometric data analysis [38]. *AO* is also capable of interacting with graphene-like materials, transforming its existing sp^2^ into sp^3^ bonding. This leads to buckling of the surface and reactions along the grain boundary fragments of the graphene layer [54]. Theoretical predictions suggest that oxygen is energetically favourable to bond on bridge sites of graphene comparing to other sites. This forms an epoxy group and further oxidation induce higher strain in the epoxy rings. It results in breaking of the C–C bonds, with the process described as oxygen-driven unzipping of graphene. It was further demonstrated that the epoxy pairs convert into carbonyl groups, leading to the fracture of the graphene sheets [55].

**Figure 8:**
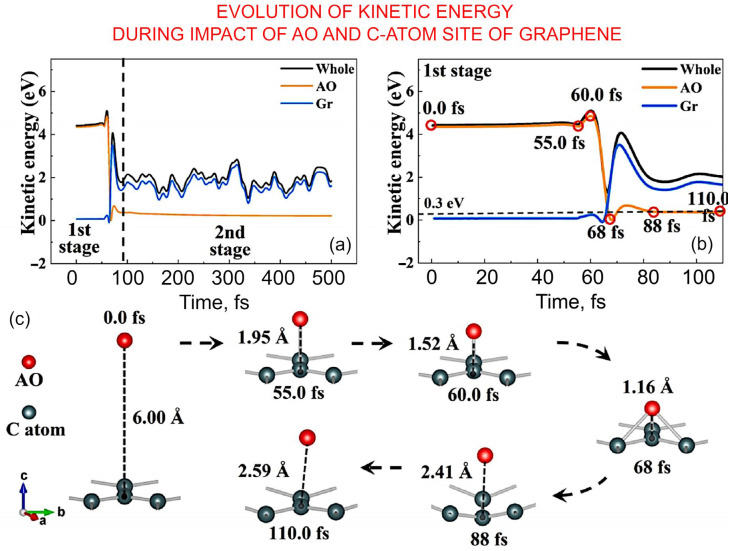
Interaction of *AO* with carbon: understanding the mechanism via simulations. (**a,b**) Evolution of kinetic energy during the collision between *AO* and *C* atom site of graphene could be split to the three stages. (**c**) Snapshots of several points marked in (**b**). Reprinted with permission from Li et al., 2023 [56] © 2022 Elsevier.

Li et al. performed a very detailed analysis of the *AO* interaction with carbon atoms, bonds, rings, etc (Figure 8). They described a two-stage process, with the initial stage taking place over the first 0 fs to 110.0 fs from impact, characterised by the movement of *AO* towards the *C* atom, followed by its scattering after it interacts with the *C* atom. The later stage involves the movement of the *AO* away from the surface, with the path being opposite to that of the incident path, with the energy of *AO* being low, at 0.30 eV.

Goto et al. conducted a series of experiments to identify the correlation between the chemical structure of various space polymers and the *AO* flux distribution. They concluded that the chemical structure is the dominant factor for the morphological changes during *AO* erosion over the mean velocity of the flux [50].

Several theoretical approaches were proposed to determine synergetic effects to correlate mass loss in LEO environment. Chen et al. constructed a unified model which can be used to evaluate the non-linear mass loss in nanocomposites. The analytic solution of mass changing rate before and after the infimum point can be given by following equations [57]:

$$\frac{M_{t}^{\left(1\right)}}{M_{0}}=\frac{{m\xi \left[PH\right]}_{0}{\left[O\right]}_{s}}{\rho }\frac{E^{\left(1\right)}}{{1+\varsigma }^{\left(1\right)}}t^{1+\varsigma^{\left(1\right)}},0\leq t<t_{inf}$$

$$\frac{M_{t}^{\left(2\right)}}{M_{0}}=\left\{\begin{array}{c} \frac{{m\xi [PH]}_{0}{\left[O\right]}_{S}}{\rho }\frac{E^{\left(2\right)}}{{1+\varsigma }^{\left(2\right)}}t^{{1+\varsigma }^{\left(2\right)}},t_{inf}\leq t<t_{cri}\\ \frac{{m\xi [PH]}_{0}{\left[O\right]}_{S}}{\rho }k_{bulk}^{extre},t\geq t_{cri} \end{array}\right.$$

where $M_{t}$, $M_{0}$, ${\left[PH\right]}_{0}$, ${\left[O\right]}_{S}$, *ρ, ξ* are mass loss, initial mass of the material, substrate concentration at the beginning, oxygen concentration, density of the material and time when an activation energy of reaction reaches its extreme [58].

## 3. Importance of Carbon Nanomaterials for the Corrosion Mitigation

Carbon materials and composites, mostly carbon fibres, are intensively used in spacecraft manufacturing for the production of, e.g., re-entry vehicle nose tips, wing leading edges, rocket nozzles and aircraft brake disks [59]. Carbon nanomaterials have exceptional mechanical, electrical and thermal properties, as well as high chemical stability, making them quite promising for protecting against corrosion in space [60]. Moreover, carbon nanomaterials can be incorporated into various matrix materials which can then be applied to metal surfaces as a coating, providing a protective barrier preventing the attachment of corrosive species [61]. The ability of carbon nanomaterials to improve the adhesion and durability of protective coatings is another factor that makes them promising for space applications where long-term performance and reliability are critical [62]. Carbon nanotubes (CNT), multiwall carbon nanotubes (MWCNT), diamond-like carbon [63,64], carbon nanorods, nanocones and nanofibers are some examples for the carbon nanomaterials used in space applications [65,66]. Below, we will discuss several specific applications of carbon nanomaterials for space materials.

### 3.1. Use of Nanomaterials for Corrosion Mitigation in Space

Coatings (metals [67], metal oxides [68,69], metal carbides [35], ceramics [70]), self-healing materials [38] and various types of surface modification [71,72] are examples of technologies that could be adapted for the mitigation of corrosion on satellites. However, coatings could be damaged by micrometeorites, space debris and handling. Additionally, internal factors such as density differences and thermal expansion may cause coating defects [73]. *AO* can penetrate through the protective coatings and damage the internal layers. Materials such as SiO_2_ can efficiently act against *AO* corrosion, but the lack of conductivity causes electrostatic build-up on SiO_2_-based materials. Electrically conductive coating materials such as indium tin oxide is an alternative, but the limited flexibility and brittle nature is an issue [39]. The use of boron nitride films as a substitute for graphene was demonstrated, with the graphene-like chemical and physical properties proven, but electrical conductivity was low [74,75]. MoS_2_ is a commonly used solid lubricating material used in many space missions including the ESA Infrared Space Observatory and James Webb Space Telescope [76] to prevent cold welding that arises as a result of friction between moving parts; however, this material is also susceptible for *AO*-induced oxidation and degradation. Efforts were made to mitigate the effects of *AO* by making highly ordered nanosheet composite of MoS_2_ [77] and, e.g., by the use of montmorillonite and poly(p-aminostyrence) against *AO* erosion on Kevlar fibres [70]. Nevertheless, the above materials do not ensure the necessary level of efficiency; thus, carbonous nanomaterials are currently under investigation due to their attractive properties such as thermal and oxidation resistance, high tensile strength-to-weight ratio and high tensile modulus-to-weight ratio. Carbon nanomaterials demonstrated excellent results as composite materials on both simulated and in situ experiments.

### 3.2. Carbon Nanotubes

Carbon nanotubes are among the most extensively studied carbon-based nanomaterial for space applications since their discovery in 1991 [78]. Abbe et al. conducted an in situ study by simulating the influence of proton, electron and gamma irradiation on CNTs and reported no significant structural changes [79]. Misak et al. conducted a thermal and *AO* study on CNT yarns and compared the results with highly oriented pyrolytic graphite. Despite the fact that the tenacity of the CNT yarns decreased with the addition of more yarns, it showed better space durability compared to pyrolytic graphite and graphite composites. However, 7% carbon depletion was observed with 18% electrical conductivity loss, indicating the effect of physically damaged CNT not only affect the mechanical properties but also negatively impacting the continuous electrical conducting pathways which carry electricity [80]. Reactive molecular dynamics simulation conducted by Rahmani et al. on CNT and graphene showed the relationship between nanotube orientation and the extent of *AO* damage. Randomly oriented CNT or graphene nanoparticles demonstrated better results in terms of lower mass loss, erosion yield, surface damage and penetration depth [81].

**Figure 9:**
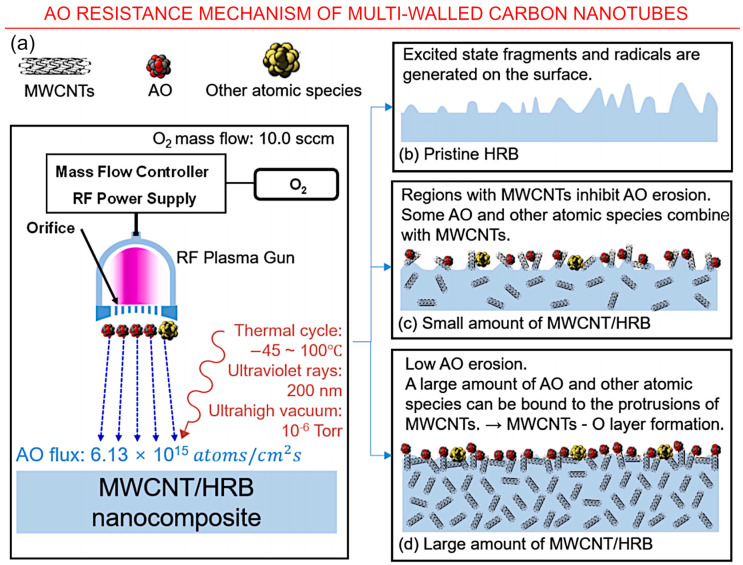
*AO* resistance mechanism of multiwalled carbon nanotubes. (**a**) Scheme of the test in space environment; (**b**) pristine hydrogen-rich benzoxazine; (**c**) small amount of multiwalled carbon nanotubes/hydrogen-rich benzoxazine, and (**d**) large amount of multiwalled carbon nanotubes/hydrogen-rich benzoxazine. Reprinted with permission from Cha et al., 2022 [85] © 2022 Elsevier.

The feasibility of employing CNT as a filler material in polybenzimidaole (PBI)-based epoxy composites were investigated by Kumar et al. It was demonstrated that a 2 wt% addition of CNT composite improved shielding performance by 50.13%. Further mechanical interlocking of the coating material and resistance to thermal cycles was also improved [82]. The self-healing property of silane with *AO* was employed by Jin et al. to prepare an *AO*-resistant MWCNT composite. Silicone reacted with *AO* and formed a SiO_2_ layer which protected the nano carbon. Furthermore, siloxane bond strengths (~8 eV) were much higher than the average energy of *AO* (~5 eV), which prevented the *AO*-induced bond from breaking [83]. Atar et al. developed a nano composite based on CNT and polyhedral oligomeric silsesquioxane (POSS) for the protection of polyimide films. The resulting film reduced the *AO* erosion by roughly one order of magnitude [84]. Figure 9 shows the erosion yields and sheet resistivity of these nanocomposites as a function of atomic oxygen fluence.

**Figure 10:**
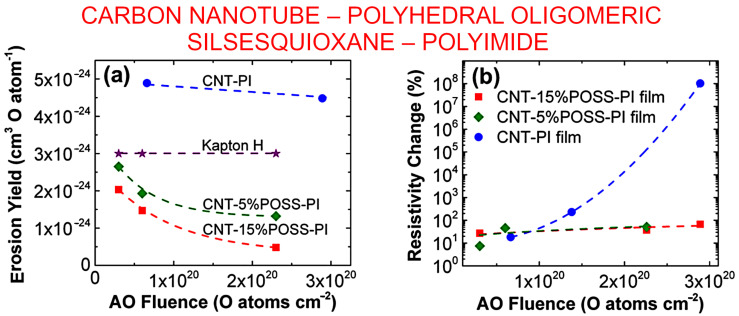
Influence of atomic oxygen exposure on carbon nanotube–polyhedral oligomeric silsesquioxane–polyimide film. (**a**) Erosion yields of the composite films and Kapton H as a function of atomic oxygen fluence. (**b**) The response of sheet resistivity of the composite films to changes in atomic oxygen fluence. Reprinted with permission from Atar et al., 2021 [85]. © ACS.

A multifunctional nanocomposite with radiation shielding properties was synthesized by Cha et al. by incorporating amine-modified MWCNT with benzoxazine. The hydrogen atoms of benzoxazine acted as the radiation shield material while MWCNT provided the mechanical strength. The composite demonstrated a 25% decrease in the total mass loss, 20% increase in tensile strength, 36% increase in tensile modulus, and it improved glass transition temperatures. This resulted in a 5.9–6.4% reduction in shielding mass comparative to the epoxy resin [75]. Figure 10 illustrates the possible *AO* resistance mechanism of multiwalled carbon nanotubes.

### 3.3. Graphene

**Figure 11:**
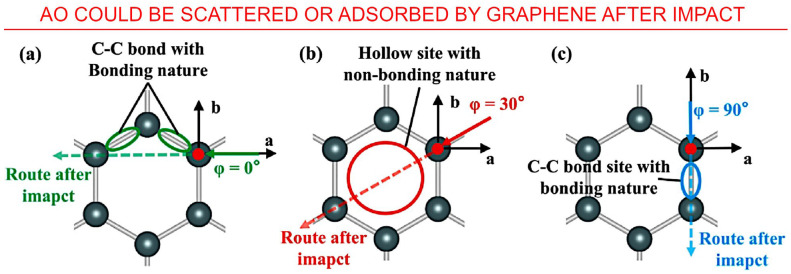
Understanding the interaction of *AO* with graphene: Schematic of the bonding conditions of the sites that *AO* fly by after the impact on *C* atom site with φ values (**a**) 0°, (**b**) 30°, (**c**) 90°. *AO* could be both scattered and adsorbed by graphene after impacting the *C* atom site. Reprinted with permission from Li et al., 2023 [56] © 2019 Elsevier.

Since its discovery in 2004, space applications of graphene included multifunctional coating materials and as communication and thermal control systems [86]. Li et al. used ab-initio molecular dynamics (AIMD) simulations to investigate the impact interactions of graphene with *AO*. The impact results showed a correlation between the extent of the damage with the impact sites and incident angle. Ring hollow site and some angles on the *C* atom site either rebound or scatter the impacting *AO*. Hence, the authors proposed the vertically faced graphene for enhanced impact protection [56]. The data are listed in Table 2 and illustrated in Figure 11.

**Figure 12:**
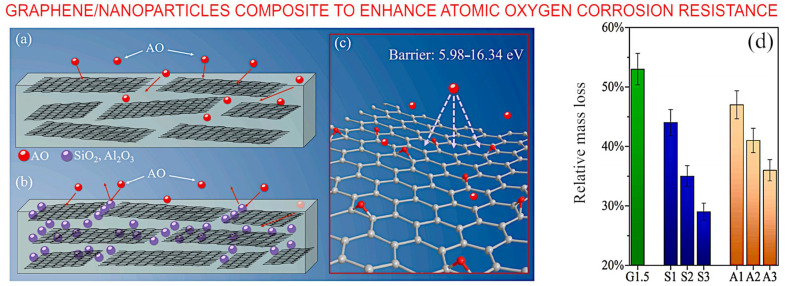
Graphene/SiO_2_ nanoparticles composite to enhance atomic oxygen corrosion resistance. (**a**) Schematic of the mechanism that (**a**) graphene flakes and (**b**) graphene flakes/SiO_2_ nanoparticles composite mitigate atomic oxygen corrosion of polyimide. (**c**) Barrier and bonding effects between graphene flakes and atomic oxygen. (**d**) Mass loss of graphene flakes/SiO_2_ nanoparticles composite film after atomic oxygen exposure. Reprinted with permission from Zhao et al., 2021 [88] © 2021 Elsevier.

The effects of *AO*, electrons and protons exposure on graphene were studied by Wang et al. Minimum damage was observed due to high energy electrons because of the electrical conductivity of graphene sheets. It was found that high energy protons can form polymorphic atomic defects via collision and excitation effects [54]. Another technique to mitigate *AO* effect on graphene sheet is changing the electronic density on the surface by doping heteroatoms. Ren et al. doped graphene sheets with nitrogen atoms and demonstrated their barrier properties experimentally and via DFT calculations [55]. Zhang et al. showed that the multilayer graphene is more effective than single layer graphene in an *AO* environment [87]. A graphene polyimide composite was synthesized by Zhao et al. An amount of 1.5 wt% of graphene reduced the mass loss by 47%. Co-doping of the composite with 3 wt% of SiO_2_ nanofiller further decreased the mass loss to 71% [88]. Figure 12 illustrates this promising graphene/SiO_2_ nanoparticles composite which is also capable to enhance atomic oxygen corrosion resistance. 

### 3.4. Carbon Quantum Dots (CQDs)

Carbon quantum dots (CQDs) are another form of carbon nanomaterials that were studied for their potential to provide corrosion resistance under different conditions. CQDs can be incorporated into polymer-based coatings to enhance their mechanical properties and provide a barrier against corrosive species. Additionally, CQDs can act as electron acceptors and help mitigate electrochemical corrosion. The effect of nitrogen-doped carbon nanomaterials in resisting corrosion were studied by Xu et al. and Gao et al. [89,90]. These studies suggested that the N-CQDs were shown to improve the performance of protective coatings by inhibiting both cathodic and anodic reactions in the corrosion process. The nitrogen atoms in the quantum dots affect the electronic structure of the metal surface, altering the reaction pathways and reducing the rate of corrosion. Additionally, the nitrogen-doping of CQDs was shown to improve their stability and reduce the risk of leaching, which is important in applications where long-term stability is required [87,91]. Interestingly, graphene could be promising for solar sails [92].

### 3.5. Graphene Oxide and Reduced Graphene Oxide

**Figure 13:**
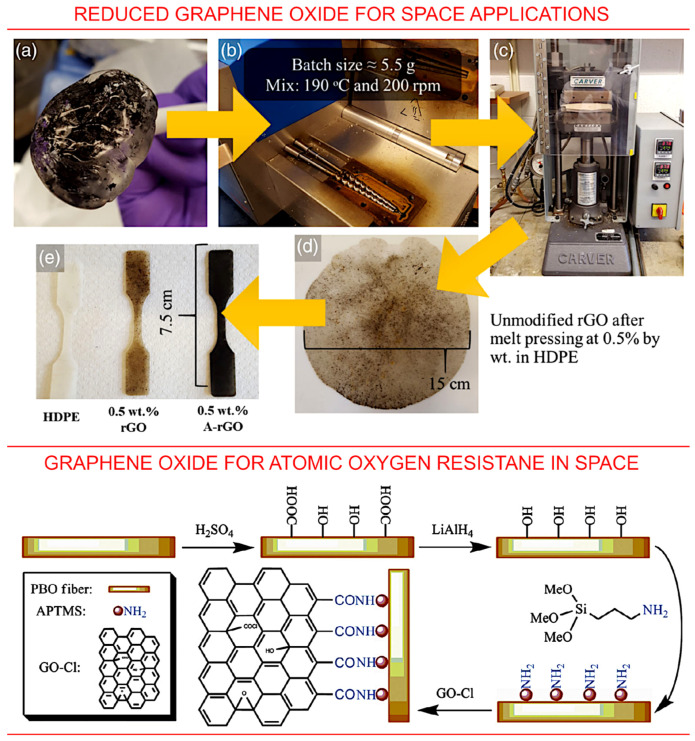
Graphene oxide and reduced graphene for space applications. Top panel: Schematics of the process used for composite fabrication, consisting of premixing (**a**), melt compounding (**b**), melt pressing (**c**,**d**) and, finally, forming of the samples (**e**). Reprinted with permission from Seibers et al., 2021 [93]. © 2019 Society of Plastics Engineers. Bottom panel: hierarchical reinforcement by grafting graphene oxide onto poly(*p*-phenylene benzobis-oxazole fibres for resistance to *AO*-induced degradation in space. Reprinted with permission from Chen et al., 2015 [95]. © 2015 Elsevier.

Graphene oxide and reduced graphene oxide are two other important examples of carbonous materials that are currently under investigation for space applications, including for the protection of low-orbit space assets from atomic oxygen, ultra-violet and space radiation [93]. The exceptional strength of graphene-based materials, along with the exceptional specific strength (i.e., the strength related to the specific density of the material) make graphene-based materials an attractive candidate for space applications. An example of an investigation of reduced graphene oxide (rGO), i.e., the processed graphene oxide with the reduced content of oxygen, is illustrated in Figure 13, top panel. Here, reduced graphene oxide was chemically functionalized and investigated for space application in the form of an additive to composites. The authors reported a decrease in electrical conductivity after the treatment; yet, rGO nanoparticles could be used to enhance the properties of composites. Specifically, the authors demonstrated an enhancement in the tensile strength of a high-density polyethylene matrix, thus evidencing the applicability of rGO-enhanced polymer coatings as protection solutions [93].

The successful application of graphene oxide (GO) for the protection of space assets from *AO* was also reported [94,95]. In one example, both silane and GO were grafted onto the poly(*p*-phenylene benzobis-oxazole) fibres to enhance their resistance to atomic oxygen. The grafting of graphene oxide was made using 3-aminopropyltrimethoxysilane. The produced hybrid fibres (Figure 13, bottom panel) featured a significant enhancement in resistance against the deleterious effects of atomic oxygen exposure.

### 3.6. Carbon Fibres for Space Applications

**Figure 14:**
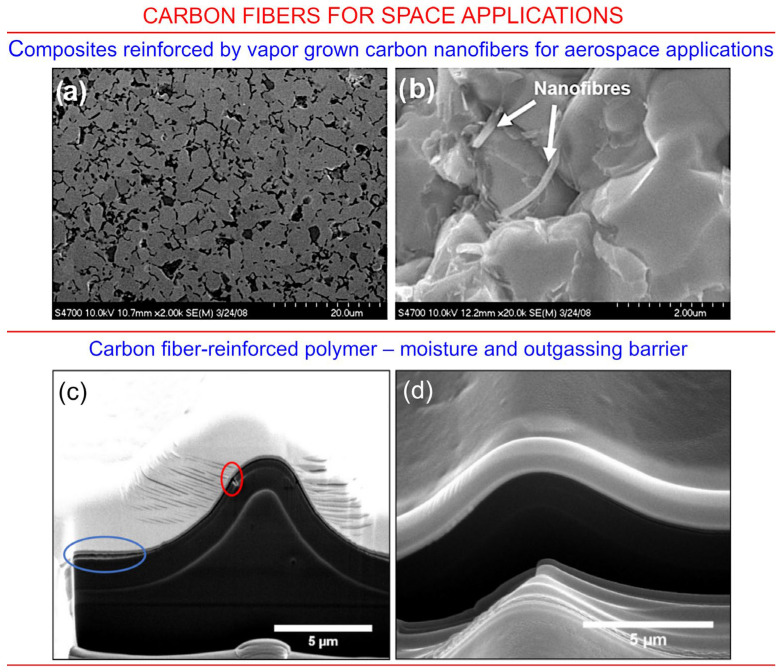
Top panel: Carbon nanofiber-reinforced composites for aerospace applications. (**a**) Illustrates the microstructure of the material after carbon coating followed with the reduction. (**b**) Surface of the fracture with the carbon nanofibers clearly visible. Reprinted with permission from Barcena et al., 2010 [96] © Wiley. Bottom panel: Atomic oxygen and UV protection for carbon fibre composite materials. (**c**) The focused ion beam was used to prepare the cross-section of the carbon fibre-reinforced polymer–moisture and outgassing barrier material system. The top protective layers delaminated due to interruption of the process in vacuum (shown in the blue ring on the photo). The red circle shows the pinholes by the atomic oxygen exposure. (**d**) same view of the focused ion beam cross-section made in the uninterrupted process, without any delaminations and holes. Reprinted with permission from Smith et al., 2021 [97] © 2021 ACS.

Carbon fibres and nano-fibres represent one more class of carbonaceous materials that is currently under consideration for the application in space technology and, particularly, for spacecraft protection. Figure 14a,b illustrate the use of carbon nanofiber-reinforced composites for aerospace applications, where fibres are added to enhance mechanical properties of the material and reduce the likelihood of their fracture as a result of collision impact or significant temperature fluctuations. The microstructure of the material after carbon coating followed by the reduction (Figure 14a) does not clearly confirm the presence of nanofibers, while the photograph of the surface of the fracture clearly shows carbon nanofibers reinforcing the top-most layer of the structure. It is worth noting that carbon nanofibers used for the reinforcement are not very resistant to atomic oxygen, and so, additional protection could be needed for the nanofiber-reinforced materials, as was recently demonstrated by Smith et al. [94]. In their study, a carbon fibre-reinforced polymer-based moisture and outgassing barrier material system was designed, and when uninterrupted process of fabrication was implemented, the coating without any delaminations and holes was obtained (Figure 14c,d).

## 4. Outlook

The LEO space environment is dynamic and challenging and can significantly reduce the durability of spacecraft materials [98]. In addition to ionizing radiation, high vacuum, plasma, space debris and thermal cycling, *AO* itself or the synergetic effect are the main causes for degradation effects on spacecrafts in LEO. *AO* may affect the material properties by changing the chemical, electrical, mechanical, thermal or optical properties.

Carbon nanomaterials and carbon-based nanocomposites were effectively employed for numerous applications in aerospace. This advanced carbon nanomaterial is capable of improving the mechanical strength of lightweight components and space environment resistance. Despite their impressive potential, space studies and modelling of some of the mechanisms and corrosion resistance of nano composites remain limited, and further studies are required to improve carbon nanocomposite derived solutions for future space applications.

The problem of *AO*-induced erosion is a special case for the VLEO systems. Along with the more dense atmosphere at very low altitudes, these satellites should use atmospheric gas as a propellant for orbit keeping by air-breathing systems, that is, the intake devices and internal parts of thruster will be affected by the highly energetic oxygen ions. To protect the thruster, several ways may be suggested for the future research and design efforts:

- Admixtures could be added to the air directly in the intake devices and internal parts of the thruster to make *AO* less chemically active and, thus, to reduce the erosion without the use of an additional protective material. However, it is worth noting that such additives could potentially lower the efficiency of these thrusters;
- Physical mechanisms and sub-systems could be used to neutralize oxygen by, e.g., chemical reaction with other substances producing oxides still suitable for the use as propellant, but less chemically aggressive;
- Special configurations of thrusters, such as electrodeless rotational systems [99] that prevent direct contact between electrodes and discharge chamber walls and oxygen plasma, could be very promising for VLEO applications;
- Self-healing materials could be promising, e.g., for super low perigee orbit satellites, where the material could self-heal during the high-altitude phase of orbit when the satellites are less affected by the exposure to atomic oxygen;
- Next, the most novel materials and nanocomposites, including advanced metallic propellants which potentially could be influenced by atomic oxygen [100], should be tested in typical VLEO environments to understand the impact of the latter;
- Finally, the ground-based facilities for the comprehensive testing of novel materials under the specific LEO and VLEO conditions need to be further designed to be more sophisticated and to accurately reflect the real conditions across their entire range. The toolkit of these technologies would significantly speed up innovation and promote the development of more diverse novel nanomaterials for space, enabled by affordable, rapid and readily available to many testing and optimisation facilities, alleviating the need for expensive in-space tests.

## Figures and Tables

**Table 1:** Polymer erosion data from the Materials International Space Station Experiment program [42].

Material	Polymer Abbreviation	MISSE 2 Mass Loss, g	Density, g/cm^3^	Area, cm^2^	MISSE 2 Erosion Yield, cm^3^/atom
Acrylonitrile butadiene styrene	ABS	0.033861	1.05	3.4944	1.09 × 10^−24^
Cellulose acetate	CA	0.191482	1.2911	3.4831	5.05 × 10^−24^
Poly-(p-phenylene terephthalamide)	PPD-T (Kevlar)	0.026790	1.4422	3.5099	6.28 × 10^−25^
Polyethylene	PE	0.102760	0.918	3.5489	>3.74 × 10^−24^
Polyvinyl fluoride	PVF (Tedlar)	0.132537	1.3792	3.5737	3.19 × 10^−24^
Crystalline polyvinylfluoride w/white pigment	PVF (White Tedlar)	0.004714	1.6241	3.4176	1.01 × 10^−25^
Polyoxymethylene; acetal; polyformaldehyde	POM (Delrin)	0.378378	1.3984	3.5119	9.14 × 10^−24^
Polyacrylonitrile	PAN	0.047281	1.1435	3.4768	1.41 × 10^−24^
Allyl diglycol carbonate	ADC (CR-39)	0.267295	1.3173	3.5392	>6.80 × 10^−24^
Polystyrene	PS	0.115947	1.0503	3.5043	3.74 × 10^−24^
Polymethyl methacrylate	PMMA	0.194588	1.1628	3.5456	>5.60 × 10^−24^
Polyethylene oxide	PEO	0.066395	1.1470	3.5591	1.93 × 10^−24^
Poly(p-phenylene-2 6-benzobisoxazole)	PBO (Zylon)	0.056778	1.3976	3.5526	1.36 × 10^−24^
Epoxide or epoxy	EP	0.140720	1.1150	3.5576	4.21 × 10^−24^
Polypropylene	PP	0.072357	0.907	3.5336	2.68 × 10^−24^
Polybutylene terephthalate	PBT	0.036429	1.3318	3.5619	9.11 × 10^−25^
Polysulphone	PSU	0.105948	1.2199	3.5010	2.94 × 10^−24^
Polyurethane	PU	0.057227	1.2345	3.5182	1.56 × 10^−24^
Polyphenylene isophthalate	PPPA (Nomex)	0.030549	0.72	3.5626	1.41 × 10^−24^
Pyrolytic graphite	PG	0.02773	2.22	3.5703	4.15 × 10^−25^
Polyetherimide	PEI	0.126853	1.2873	3.5352	>3.31 × 10^−24^
Polyamide 6 or nylon 6	PA 6	0.118376	1.1233	3.5646	3.51 × 10^−24^
Polyamide 66 or nylon 66	PA 66	0.065562	1.2252	3.5249	1.80 × 10^−24^
Polyimide	PI (CP1)	0.080648	1.4193	3.5316	1.91 × 10^−24^
Polyimide (PMDA)	PI (Kapton H)	0.124780	1.4273	3.4590	3.00 × 10^−24^
Polyimide (PMDA)	PI (Kapton HN)	0.121315	1.4346	3.5676	2.81 × 10^−24^
Polyimide (BPDA)	PI (Upilex-S)	0.038127	1.3866	3.5382	9.22 × 10^−25^
Polyimide (PMDA)	PI (Kapton H)	0.129250	1.4273	3.5773	3.00 × 10^−24^
High temperature polyimide resin	PI (PMR-15)	0.118887	1.3232	3.5256	>3.02 × 10^−24^
Polybenzimidazole	PBI	0.082708	1.2758	3.4762	>2.21 × 10^−24^
Polycarbonate	PC	0.142287	1.1231	3.5010	4.29 × 10^−24^
Polyetheretherkeytone	PEEK	0.107764	1.2259	3.4821	2.99 × 10^−24^
Polyethylene terephthalate	PET (Mylar)	0.125187	1.3925	3.5432	3.01 × 10^−24^
Chlorotrifluoroethylene	CTFE (Kel-f)	0.052949	2.1327	3.5452	8.31 × 10^−25^
Ethylene-chlorotrifluoroethylene	ECTFE (Halar)	0.088869	1.6761	3.5103	1.79 × 10^−24^
Tetrafluorethylene-ethylene copolymer	ETFE (Tefzel)	0.049108	1.7397	3.4854	9.61 × 10^−25^
Fluorinated ethylene propylene	FEP	0.012479	2.1443	3.4468	2.00 × 10^−25^
Polytetrafluoroethylene	PTFE	0.008938	2.1503	3.4841	1.42 × 10^−25^
Perfluoroalkoxy copolymer resin	PFA	0.010785	2.1383	3.4570	1.73 × 10^−25^
Amorphous Fluoropolymer	AF	0.012352	2.1463	3.4544	1.98 × 10^−25^
Polyvinylidene fluoride	PVDF (Kynar)	0.066860	1.7623	3.4993	1.29 × 10^−24^

**Table 2:** Impact interaction results between the *AO* and graphene with various incident angles and impact sites [56].

Impact Sites	Incident Angle φ, °	Incident Angle θ, °	Impact Interaction Results
Ring hollow	0°, 30°	45°, 60°, 75°	Rebounded
C–C bond	0°, 30°,90°	45°, 60°, 75°	Adsorbed
*C* atom	0°, 90°	45°, 60°, 75°	Rebounded first and then adsorbed on C–C bond
	30°	45°, 60°, 75°	Rebounded

## Data Availability

No new data were created.
